# A Review of Anodized TiNbSn Alloys for Improvement in Layer Quality and Application to Orthopedic Implants

**DOI:** 10.3390/ma15155116

**Published:** 2022-07-22

**Authors:** Yu Mori, Naoya Masahashi, Toshimi Aizawa

**Affiliations:** 1 Department of Orthopaedic Surgery, Tohoku University Graduate School of Medicine, 1-1 Seiryo-machi, Aoba-ku, Sendai 980-8574, Japan; toshi-7@med.tohoku.ac.jp; 2 Institute for Materials Research, Tohoku University, 2-1-1 Katahira, Aoba-ku, Sendai 980-8577, Japan; naoya.masahashi.e6@tohoku.ac.jp

**Keywords:** anodic oxide, antibacterial activity, orthopedic implants, osseointegration, photocatalyst, TiNbSn alloy

## Abstract

Titanium alloys are useful for application in orthopedic implants. However, complications, such as prosthetic infections and aseptic loosening, often occur after orthopedic devices are implanted. Therefore, innovation in surface modification techniques is essential to develop orthopedic materials with optimal properties at the biomaterial–bone interface. In this review, we present recent research on the improvement in the osteoconductivity and antibacterial effect of the Ti-33.6% Nb-4% Sn (TiNbSn) alloy by anodic oxidation and other related studies. TiNbSn alloys are excellent new titanium alloys with a low Young’s modulus, high tensile strength, and with gradient functional properties such as a thermally adjustable Young’s modulus and strength. Titanium dioxide (TiO_2_), when obtained by the anodic oxidation of a TiNbSn alloy, improves bone affinity and provides antibacterial performance owing to its photocatalytic activity. The safety of TiO_2_ and its strong bonding with metal materials make its method of preparation a promising alternative to conventional methods for improving the surface quality of orthopedic implants. Implementing anodization technology for TiNbSn alloys may alleviate orthopedic surgery-related complications, such as loosening, stress shielding, and infection after arthroplasty.

## 1. Introduction

Some afflictions that worsen with age are osteoarthritis, joint pain, and functional disorder caused by age-related degeneration of the articular cartilage in the knee and hip joints [1]. The hip joint, which is the largest joint in the human body, supports the body weight and is responsible for various movements of the body. With an increase in age, in addition to severe pain and muscle weakness, the ability to walk is also impaired owing to the shortening of the lower limb length, resulting in a significant limitation of daily activities. Consequently, the number of total hip arthroplasties (THAs) for osteoarthritis is increasing worldwide [2]. 

THA consists of a stem placed in the femur, a head placed over the stem, a cup placed in the acetabulum, and a liner that serves as a joint surface. The stem and cup are usually made of metal, the head of metal or ceramic, and the liner of polyethylene. The stem is typically composed of titanium alloy. Favorable long-term results of THA have been reported in patients with hip osteoarthritis. Previous long-term follow-up studies have shown that improvements in hip pain, function, and quality of life were achieved by THA [3,4]. Furthermore, the long-term results of cementless and cemented femoral stems have shown favorable results in clinical and radiological evaluations [5,6,7]. Therefore, THA with cementless stems is gaining worldwide popularity.

However, THA with cementless stems has several problems that need to be addressed. One major problem is stress shielding owing to improper load distribution between the femur and stem. Stress shielding refers to bone atrophy and loss of bone density due to the load distribution being removed from the bone by the femoral stem [8,9,10]. Bone atrophy and loss of bone density due to stress shielding increase the risk of periprosthetic fractures [11,12]. With the increasing number of THA procedures performed in elderly patients with osteoporosis, preventing periprosthetic fractures is very important. The etiology of stress shielding is thought to be multifactorial, including the stem surface texture, shape, and stiffness [13]. Currently, femoral stems are made of Ti-6Al-4V alloy with a Young’s modulus of 110 GPa and tensile strength of 860 MPa; the Ti-6Al-4V alloy has good biocompatibility and excellent corrosion resistance. In contrast, the Young’s modulus of the human cortical bone ranges from 10 GPa to 30 GPa [14]. This mismatch in elasticity between the femoral stem and cortical bone is considered one of the main causes of stress shielding. There is an urgent need for metallic materials to have sufficient strength with an elastic modulus close to that of bone.

**Figure 1:**
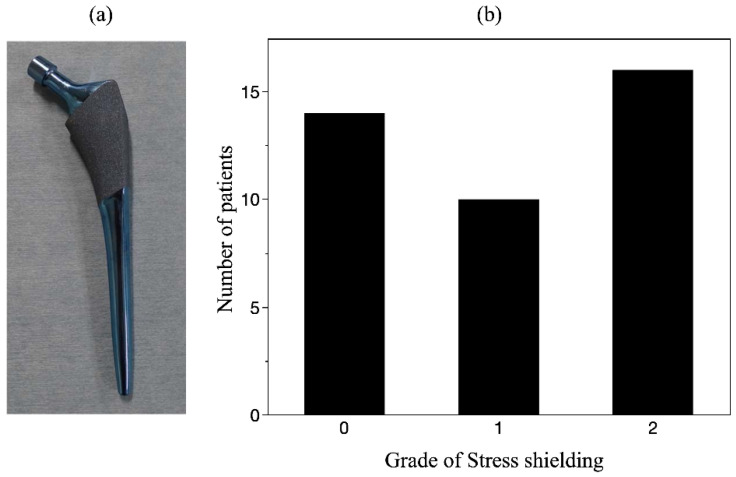
**Clinical application of TiNbSn femoral stem:** (**a**): Photograph of TiNbSn femoral stem, (**b**): Mid-term results at 3 years after surgeries show the incidence of only mild stress shielding. Engh’s stress shielding classification is grade 0 to 4, with 5 levels. No serious stress sealing of grade 3 or higher was observed.

Hanada et al., developed a new β titanium alloy with a Young’s modulus as low as 40 GPa, Ti-33.6% Nb-4% Sn (TiNbSn) alloy, which is characterized not only by its low Young’s modulus but also by its ability to control mechanical properties by heating. In this TiNbSn alloy, the alloy with a near-β-phase composition is subjected to groove rolling and swaging to stabilize the α″phase and align the β<110>parallel to the α″<010>orientations. Additionally, safety and biocompatibility have been demonstrated in previous studies [15,16]. Our research group developed a new cementless stem of TiNbSn alloy with functional gradient properties of Young’s modulus and strength upon heat treatment [16]. The stem shape is then machined, and the proximal portion is locally heated without changing the low Young’s modulus of the distal portion to produce a stem with both a low Young’s modulus and high strength by increasing the strength through micro-precipitation of the α-phase. TiNbSn alloy stems with high proximal strength and low Young’s modulus in the distal part are considered ideal for fatigue fracture prevention and stress shielding. In the mid-term results of clinical trials of TiNbSn alloy stems, stress shielding was reported to be suppressed [17] (Figure 1). The 3-year post-operative results of the TiNbSn stem clinical trial showed only minor stress shielding of grade 2 or less on Engh’s classification of stress shielding [10,17]. This is the first report of clinical trial results of a low Young’s modulus titanium alloy hip prosthesis, and TiNbSn alloy is a high-performance material that can be clinically applied as an orthopedic implant. Furthermore, it has been reported that the low elastic modulus of TiNbSn alloys may be useful for promoting fracture healing. Previous reports have also shown that the healing of tibial fractures in mice and rabbits is accelerated when intramedullary nails of TiNbSn alloy are used compared with Ti6Al4V alloy or stainless steel [18,19,20,21]. Fracture healing mechanisms are multifaceted, and studies have shown that fracture repair requires the promotion of differentiation of stem cells on the periosteum into osteoblasts and the induction of appropriate inflammation [22,23]. Similar to these mechanisms, the mechanism for accelerated healing by the TiNbSn alloy is studied as a proper load distribution between the bone and the fracture fixation device, which promotes osteoblast differentiation and bone formation at the fracture site.

In this review, we introduce recent research on the improvement in osteoconductivity and the antibacterial property of TiNbSn alloys and other related studies. In the case of cementless stems, osseointegration is essential, but since metal has no function in inducing osteogenesis, surface roughening by blasting and laser irradiation, coating with hydroxyapatite (HAp), which is the main component of bone, and alkaline treatments such as sodium hydroxide are applied to the stem surface for the purpose of osteogenesis [24]. From the viewpoint of adverse effects of metal ions on cells [25], surface roughening that easily leaches metal ions is undesirable. Even metals that do not exhibit cytotoxicity may be harmful to cells, and exposure to metal surfaces should be avoided, if possible. On the other hand, HAp coatings do not provide sufficient osteoconductivity owing to HAp modification during the coating process [26] and poor adhesion to the metal [27]. Alkali treatment requires heat treatment at approximately 600 °C and cannot be applied to TiNbSn alloys because it causes an increase in Young’s modulus due to phase precipitation and reverses the transformation.

Bone is a porous structure that promotes cell fixation and proliferation and is formed from oxides such as calcium phosphate and calcium carbonate, with calcium (Ca), phosphorus (P), magnesium (Mg), oxygen (O), and other elements as major components. These oxides are known to promote biocompatibility of implant materials [28], and oxide coatings that are in equilibrium with bone-constituting elements and have high adhesiveness to metals are effective for osteoconductivity. The authors focused on titanium dioxide (TiO_2_), a passive oxide of titanium that is thermodynamically equilibrated with these oxides (CaTiO_3_, P_2_O_5_, MgO_2_, etc.) and is considered to exhibit high adhesion to titanium alloy substrates. The authors considered that TiO_2_ on TiNbSn alloy by anodic oxidation could promote osseointegration by improving the biocompatibility and could also provide antibacterial activity by photocatalytic property. In addition to the low Young’s modulus of the TiNbSn alloy hip prostheses, the improvement in osteoconductivity by anodic oxidation may be more effective in preventing stress shielding. In addition, if it is possible to add antibacterial properties to joint prostheses to prevent infection, which is an issue in joint replacement surgery, it could make a significant contribution to improving the long-term performance of joint replacement surgeries. In this review, we describe the findings of anodic oxide on TiNbSn alloy substrates to provide osteoconductivity and photocatalytic activity to TiNbSn alloys and to verify the biocompatibility of the TiO_2_ coating.

## 2. Anodic Oxidation

Anodic oxidation is a method of forming oxides or insoluble layers on metal surfaces using electrochemical techniques and is used to improve corrosion and wear resistance. In anodic oxidation, metal is dissolved as a cation in an electrolyte solution, where it reacts with the oxygen ions originating from the electrolysis of water to form a metal oxide. Anodized alumite, which is used industrially, is the anodic oxidation of aluminum. Columnar pores were arranged on a thin barrier layer parallel to the oxide growth direction, resulting in a honeycomb shape when viewed from the oxide growth direction. The oxide grows in proportion to the current, but at the same time, it dissolves at the bottom of the cell, and the undissolved portion grows as the cell wall, forming a pore structure. However, anodized titanium alloys have practical applications, such as being used as a coloring technique owing to the light interference effect of the oxide. Optical interference is caused by the optical path difference between the light reflected from the surface of the oxide and that from the bottom of the oxide (substrate surface) transmitted through the oxide. The preferred color varies depending on the thickness of the layer.

The pore structure of anodic oxide layers, such as aluminum oxide, has also been reported for titanium oxide [29,30]. Recently, TiO_2_ nanotubes have been widely studied as catalysts, gas sensors, and bio-templates. The fabrication of TiO_2_ nanotubes can be roughly divided into hydrothermal synthesis and anodic oxidation methods. In particular, TiO_2_ nanotubes fabricated by anodic oxidation, which are capable of self-assembly, have high strength, a large surface area, high electron mobility, and a quantum confinement effect [31]. The structure formation mechanism is based mainly on the model of oxide layer growth and dissolution accompanied by the application of electric current [32]. However, a model based on ion electromigration and viscous motion has also been proposed [33].

In vivo studies on the osteoconductivity of TiO_2_ nanotubes have reported that titanium alloys coated with TiO_2_ nanotubes exhibit higher osteoconductivity than blasted or acid-treated titanium alloy materials [34,35]. This is attributed to the formation of anatase TiO_2_ coherent with HAp and the osteoblastic differentiation and interfacial bone formation of fluorine in TiO_2_ owing to the fluoric acid in the electrolytic bath [34]. As osteoconductivity depends on the pore size of the nanotubes [34,36], the interaction of TiO_2_ nanotubes with integrins, which are protoplasmic proteins on the cell surface, and with the cytoskeleton has also been reported [37]. Furthermore, since cell proliferation requires intracellular signaling functions, it has been reported that structures growing perpendicular to the substrate promote cellular uptake [38]. Chen et al., reported that coating TiO_2_ nanotubes with anti-sclerostin antibodies promotes osteoblast differentiation and activation, inhibits the secretion of the osteoblast suppressor molecule sclerostin, and increases alkaline phosphatase activity, which in turn increases osteoblast activity [39]. Although the biocompatibility mechanism of TiO_2_ nanotubes has not been established, TiO_2_ nanotubes are attractive surface structures that provide osteoconductivity to titanium alloy implants.

In contrast, in a previous study on the biocompatibility of anodized TiO_2_, which does not have a pore structure, a high electric field was applied in a weak acid to increase surface roughness [40,41,42,43,44,45]. Sul et al., produced anodic oxide layers of pure titanium with surface roughness ranging from 0.83 to 1.02 μm by systematically changing the potential from 100 V to 380 V in a 0.1 M acetic acid electrolysis bath [40]. In vivo tests confirmed the formation of new bone on TiO_2_ and showed that a larger surface roughness promoted more bone formation [45]. Subsequently, similar studies using different potentials and electrolytic bath compositions were reported [42,43,44]. In many cases, such layer formation is accompanied by micro-arc oxidation on the electrode surface, indicating surface roughening due to dielectric breakdown.

## 3. Anodic Oxidation on Titanium and TiNbSn Alloy

TiO_2_ nanotubes reported in previous studies were anodized in a fluorine-containing electrolytic bath followed by annealing at 450–600 °C for 1–3 h [40,45]. However, heat treatment cannot be employed for TiNbSn alloys because Young’s modulus increases owing to the α-phase precipitation by reverse transformation. Heat treatment in nanotube fabrication is used to crystallize oxide layers and has been reported to suppress the recombination of excited charge carriers in TiO_2_ and increase the quantum efficiency [46,47]. Although the effect of heat treatment on photocatalytic quantum device applications is understandable, the necessity of crystallization for biomaterial applications is unclear. However, heat treatment has also been used in studies conducted on biological applications [34,35,36,48,49,50]. An anodic oxidation technology that is different from nanotube TiO_2_ technology, which requires heat treatment, is presented.

**Figure 2:**
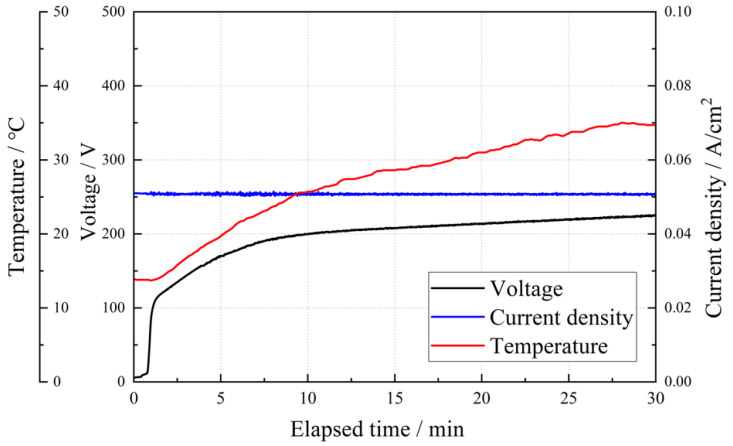
**Changes in voltage, current, and temperature during anodic oxidation.** Variation in the voltage, current density, and temperature against time during anodization in 1 M sulfuric acid electrolytes on the TiNbSn substrate.

Most TiO_2_ materials that exhibit photo-induced functions are prepared by the sol–gel method, but since it is difficult to coat TiO_2_ on large or complex-shaped substrates or substrates with poor heat resistance, the authors have been engaged in research on the photo-induced function of anodic TiO_2_. When a titanium electrode is anodized by applying an electric field of approximately 200 V in a sulfuric acid electrolytic bath, the crystal structure of TiO_2_ on the substrate gradually changes from anatase to rutile with an increase in sulfuric acid concentration in the electrolyte. The crystallinity of TiO_2_ increases with it, thus improving the photoinduced function [51]. Sulfur dissolved in TiO_2_ from the electrolyte reduces the bandgap energy of TiO_2_, thus improving the visible light response [52]. The oxidation reaction on the electrode surface is accelerated by a high electric field, and the occurrence of sparks (discharge) caused by dielectric breakdown increases the electrolyte temperature, which is more pronounced at higher sulfuric acid concentrations (Figure 2). To our understanding, the improvement in the photocatalytic function can be attributed to the decrease in the density of lattice defects, which can be the recombination sites of photogenerated charge carriers, owing to the improvement in crystallinity [51]. Sparks caused by the application of a high electric field have also been reported in 1.4 M phosphoric acid electrolysis baths; when the potential was increased from 50 to 250 V, sparks occurred at 200 V as the layer thickness increased [53]. 

## 4. Biocompatibility of Anodized TiNbSn Alloy

**Figure 3:**
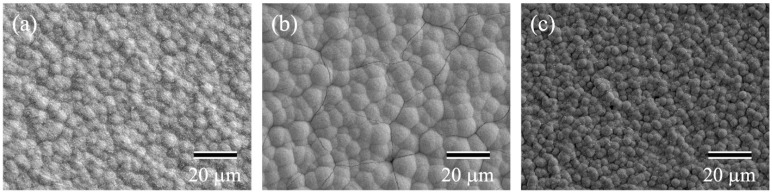
**SEM microstructures of the HW-treated anodic oxides.** Representative images of the SEM microstructures of HW-treated anodic oxides prepared in the electrolyte with acetic acid. Acetic acid concentration: (**a**) 1 M; (**b**) 2 M; (**c**) 6 M. SEM: Scanning electron microscopy; HW: Hot water. (reprinted from Ref. [57] with permission).

The anodic oxidation of titanium and its alliance substrate has been studied [54,55]. After immersion in hot water at 80 °C for 48 h, drying at 40 °C for 24 h, and immersion in artificial body fluids for 1, 3, and 7 h, HAp precipitated on the anodized specimens with hot water treatment, but not on the anodized specimens without hot water treatment. A similar experiment was conducted on a TiNbSn alloy [56]. The TiNbSn alloy substrate was anodized in a 1 M acetic acid electrolytic bath at 200 V for 30 min, immersed in 25 mL of Hanks’ solution (maintained at 36.5 °C) for 7 days, washed with distilled water, and dried in a dry incubator for 24 h. HAp was observed in the anodized alloy subjected to hot water treatment but not in the anodized alloy [57] (Figure 3). Surface analysis revealed that this microstructure was HAp; hence, bioactivity could be obtained by hot water treatment of the anodized layer on a TiNbSn substrate. 

**Figure 4:**
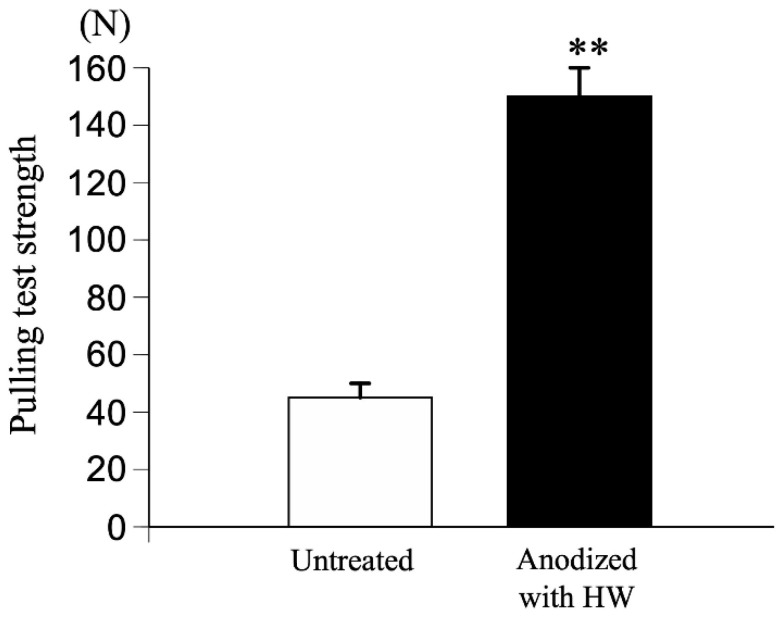
**Comparison of pull-out strength of TiNbSn rods treated by acetic acid anodization with hot water treatment and untreated TiNbSn rods**. Pull-out strength was assessed six weeks after TiNbSn rod implantation into rabbit femurs. **: *p* < 0.01 using Student’s *t* test. HW: hot water.

**Figure 5:**
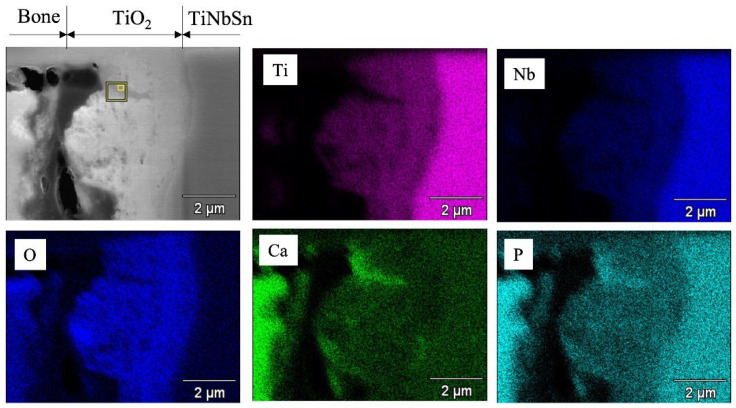
**Evaluation of bone-metal interface by SEM image and EDX mapping of the elements with implanted TiNbSn rod**. Evaluation of the bone–metal interface was performed by SEM imaging and EDX mapping of Ti, Nb, O, Ca, and P near the rabbit bone and the implanted TiNbSn rod. SEM: Scanning electron microscopy; EDX: Energy dispersive X-ray spectroscopy. (reprinted from Ref. [57] with permission).

For experimental animal models, a rod, 4.5 mm in diameter and 32 mm in length, was machined by lathe turning from a round bar of TiNbSn alloy (with a diameter of 8 mm) and a protruding part with a round hole at one end for jig mounting was fabricated for the pull-out test. The rods were anodized in a 2 M acetic acid electrolytic bath at a current density of 50 mA/cm^2^ under an applied limit of 500 V applied voltage, and a treatment time of 30 min, followed by hot water treatment. The specimens were placed in the bone marrow cavity of white rabbit femurs and maintained for 3 or 6 weeks after implantation. The bonding between the implanted alloy and the bone was investigated. The anodized alloy with hot water treatment showed higher pull-out strength than the rod without anodization, indicating that the adhesion between the implant and the bone tissue formed on the surface of the implant was strengthened by hot water treatment (Figure 4). The specimens with and without hot water treatment were prepared using a focused ion beam (FIB) from the distal and proximal parts of the femurs, and microstructure observation and energy dispersive X-ray spectroscopy (EDX) analysis were conducted using transmission electron microscopy (TEM) near the implant–bone interface [57] (Figure 5). Both Ca and P, originating from the bone, were detected in the anodic oxide layer. The high adhesion strength can be attributed to the penetration of Ca and P, which are the constituent elements of bone, into TiO_2_.

**Figure 6:**
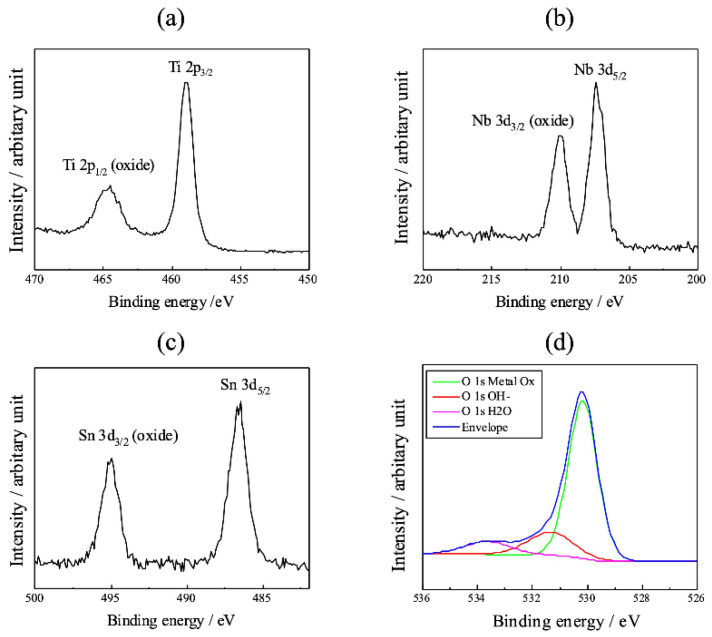
**XPS analysis of anodized TiNbSn alloy with hot water treatment**. (**a**) Ti 2p; (**b**) Nb 3d; (**c**) Sn 3d; (**d**) O 1s XPS of hot water treated anodic oxide on TiNbSn prepared in the electrolyte with 1 M acetic acid. XPS: X-ray photoelectron spectroscopy.

**Figure 7:**
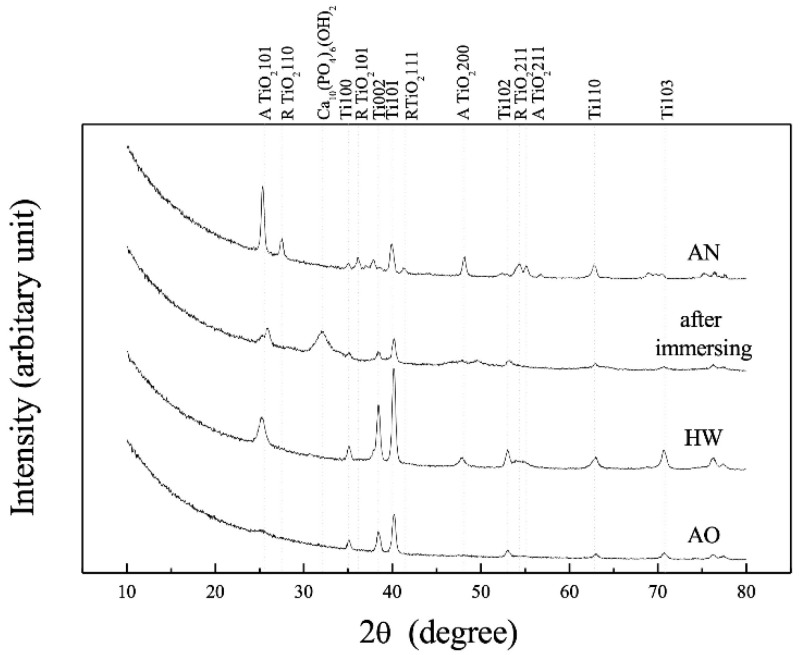
**XRD profiles of the anodized oxide prepared in 1 M acetic acid and subsequent treatment**. XRD profiles of the as-anodized oxide prepared in 1 M acetic acid electrolyte, the subsequent HW-treated oxide, and after immersion in Hank’s solution. The XRD profile of the annealed anodic oxide was used as a reference. XRD: X-ray diffraction; AN: annealed anodized oxide; AO: anodized oxide; HW: hot water-treated anodic oxide.

According to previous studies on the biocompatibility of anodized TiO_2_, the mechanism of its development differs for nanotubes and surface-roughening. Nanotubes have been proposed in terms of both the porous structure and composition of TiO_2_, although a consistent mechanism has not been established yet. However, the latter generally involves the enhancement of osteoblast adsorption by TiO_2_ surface roughening, although there are some differences among reports. The mechanism of HAp formation by hot water treatment is similar to this approach, and Kokubo et al., proposed an increase in hydroxyl adsorption by hot water treatment and crystallographic coherency between rutile TiO_2_ and HAp [54,55]. The results of XPS angle-resolved measurements of anodized TiNbSn alloys prepared in a 1 M acetic acid electrolyte have been reported [57]. After hot water treatment, XPS analysis revealed that the fraction of O 1 s hydroxyl groups decreased, and the oxide fraction increased compared to those before treatment. For niobium (Nb) oxide (Nb_2_O_5_) and tin (Sn) oxide (SnO_2_), hot water treatment increased the amount of Nb and Sn and did not promote an increase in the number of hydroxyl groups (Figure 6). On the other hand, in the thin layer X-ray diffraction profiles of TiNbSn alloy before hot water treatment, after hot water treatment, and after in vitro testing [56], the anatase 101 diffraction intensity, which was weak before hot water treatment, increased after the treatment, and after in vitro testing, HAp diffraction could be confirmed, but rutile phase diffraction was not detected (Figure 7). In other words, the effect of hot-water treatment on bone conductivity improvement may be due to factors other than the two stated above. The authors focused on the permeation of bone constituents into TiO_2_ and hypothesized that a membrane structure that facilitates the permeation of bone constituents is effective in improving bone conductivity.

**Figure 8:**
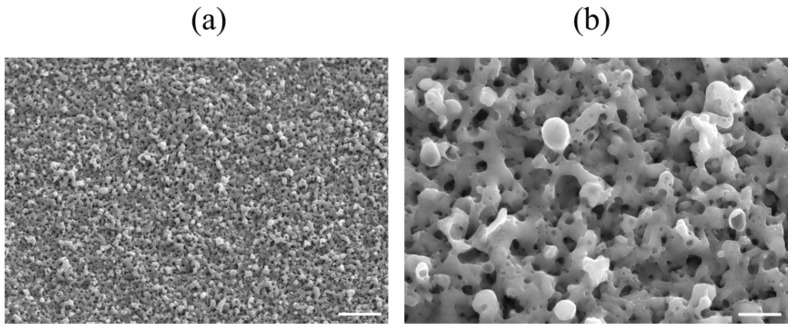
**SEM images of the anodic oxides prepared in 1 M sulfuric acid electrolyte on TiNbSn alloy**. Representative SEM images of the anodic oxides prepared in 1 M sulfuric acid electrolyte on TiNbSn alloy. (**a**) Low magnification: scale bar indicates 10.0 μm, (**b**) high magnification: scale bar indicates 2.0 μm. SEM: Scanning electron microscopy.

**Figure 9:**
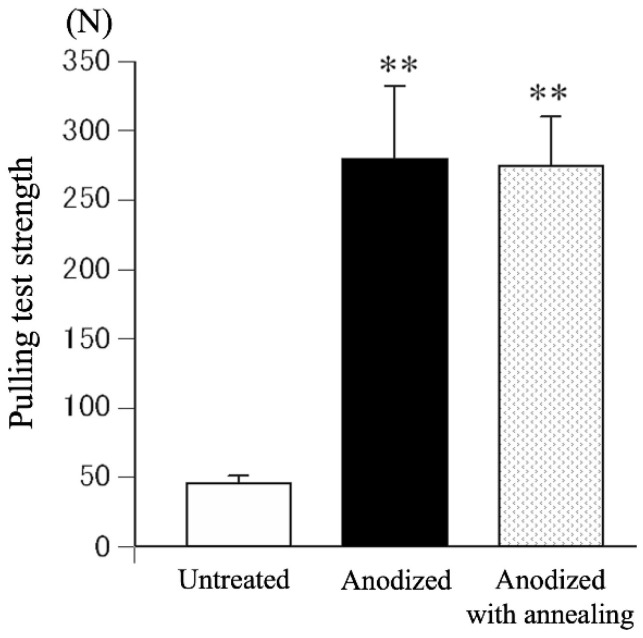
**Comparison of pull-out strength of TiNbSn rods treated by sulfuric acid anodization, sulfuric acid anodization with annealing treatment, and untreated TiNbSn rods**. The pull-out strength was assessed six weeks after TiNbSn rod implantation into rabbit femurs. **: *p* < 0.01 using one-way analysis of variance post hoc by Tukey Kramer test.

**Figure 10:**
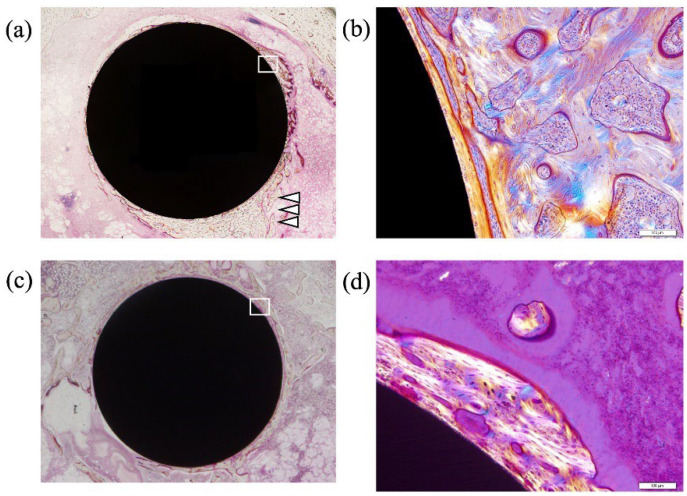
**Non-decalcified histological assessment of TiNbSn implanted femurs.** TiNbSn rods anodized with sulfuric acid, and untreated TiNbSn rods were implanted in rabbit femurs. Histological assessment was performed six weeks after TiNbSn rod implantation. Arrowheads indicate new bone formation. (**a**) Low-magnification image of anodized TiNbSn rod groups. (**b**) Low-magnification image of untreated TiNbSn rod group. (**c**) High-magnification image of anodized TiNbSn rod group. (**d**) High-magnification image of untreated TiNbSn rod group.

**Figure 11:**
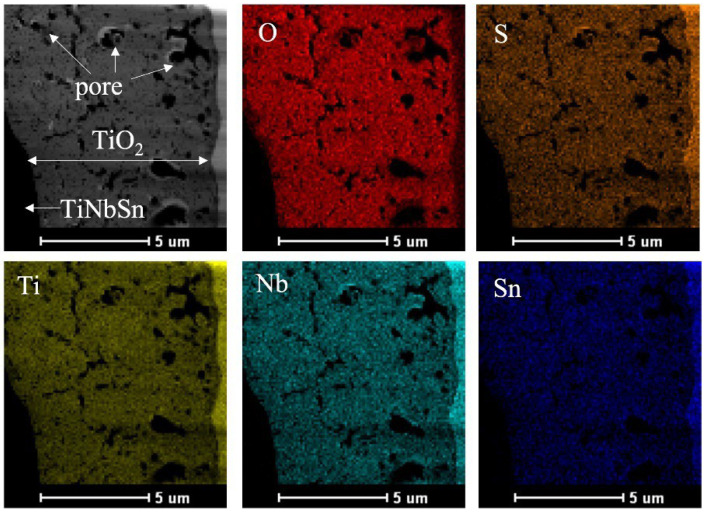
**TEM micrographs and EDX elemental mapping of the annealed anodic oxides prepared in an electrolyte with sulfuric acid**. Cross-sectional TEM micrographs and EDX elemental mapping of annealed anodic oxides prepared in an electrolyte with a sulfuric acid concentration of 1.2 M. TEM: Transmission electron microscope; EDX: Energy dispersive X-ray spectroscopy.

The electrolytic bath for anodic oxidation was changed from acetic acid (weak acid) to sulfuric acid (strong acid) and similar experiments were conducted to obtain a porous structure induced by the generation of oxygen molecules. Anatase TiO_2_ appeared in the anodic oxide prepared in the electrolyte of 1 M acetic acid aqueous solution, while rutile TiO_2_ was observed in 1 M sulfuric acid aqueous solution. The surface roughness Ra was 2.0 μm for TiO_2_ prepared in the 1 M acetic acid electrolysis bath and was 1.7 μm after hot water treatment; on the other hand, it was 2.3 μm for TiO_2_ prepared in the 1 M sulfuric acid electrolysis bath and was 2.0 μm after hot water treatment. The surface area of TiO_2_ anodized in a 1 M sulfuric acid electrolysis bath was approximately 30% larger than that of TiO_2_ anodized in a 1 M acetic acid electrolysis bath. Cross-sectional TEM observations showed that the layer anodized with 1 M acetic acid was approximately 370 nm thick, but the layer anodized with 1 M sulfuric acid was approximately 7.7 μm thicker, and the frequency of internal pores was significantly increased. In vitro tests on anodized TiNbSn alloy prepared in a sulfuric acid electrolyte with or without hot water treatment detected HAp in the hot water-treated oxide, but HAp was not detected in the untreated oxide (Figure 8). In vivo tests showed that the pull-out strength of the hot-water-treated anodized TiNbSn alloy was higher than that of the TiNbSn alloy anodized in an acetic acid electrolytic bath. The results of the bone bonding strength test showed that the pull-out strength of the TiNbSn alloy anodized with sulfuric acid was higher than that of the untreated rod (Figure 9). Furthermore, the pull-out strength was equally high regardless of the hot water or heating treatment [58,59]. Non-decalcified tissue images of untreated and anodized rods were shown after six weeks of implantation in a rabbit femur. The anodized group exhibited vigorous bone formation around the specimens (Figure 10). The bone–TiNbSn alloy interface was sampled using a FIB and mapped by EDX, and the results showed that there was no P or Ca in TiO_2_. The results of EDX mapping of the bone–TiNbSn alloy interface revealed that P and Ca permeated into TiO_2_, especially in the pores, indicating that P and Ca were concentrated in TiO_2_ (Figure 11). This result suggests that the pores in TiO_2_ are open pores that connect the surface to the interior, but it may also be related to the fact that Ca and P can be solid soluble in TiO_2_ [60,61,62,63,64]. Oxygen in the hydroxyl ions, which originate from the water in the electrolytic bath, is supplied to form the oxide layer and remains in the layer as oxygen molecules to form pores [65,66]. When TiO_2_-containing pores are immersed in Hanks’ solution, Ca and P ions constituting Hanks’ solution adsorb and permeate into TiO_2_. Some permeated ions dissolve solidly, and some accumulate in pores within TiO_2_. In contrast, a TiO_2_ surface with high roughness becomes hydrophilic according to Wenzel’s theory [67], and the adsorbed Ca, P, and hydroxyl ions form HAp. The accumulation of Ca and P ions in the pores is accelerated with their solid dissolution in TiO_2_, and the HAp grows. Not all porous materials with large surface roughness show osteoconductivity, and chemical interactions with substances in body fluids, as proposed by Kokubo [24] and others, are indispensable. Considering the biocompatibility of oxides, TiO_2_ coating is effective for the osteoconductivity of TiNbSn alloys, and the mechanism of its expression should be considered from both the function and structure of the surface layer.

## 5. Photocatalytic Activity of Anodized TiNbSn Alloy

**Figure 12:**
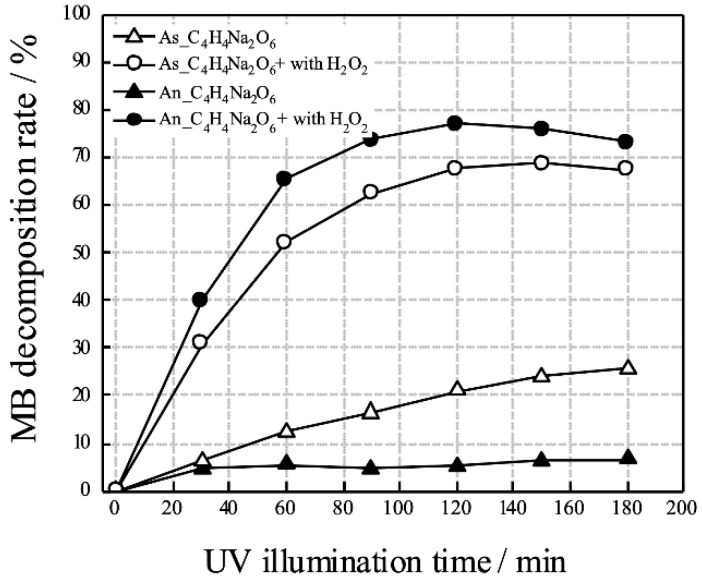
**Degradation rate of MB against UV illumination time for anodized TiNbSn alloy prepared in sodium tartrate.** Plots of the degradation rate of MB against UV illumination time for the as-anodized and annealed anodic oxides prepared in sodium tartrate electrolyte with or without H_2_O_2_ on the TiNbSn alloy. MB: Methylene blue.

**Figure 13:**
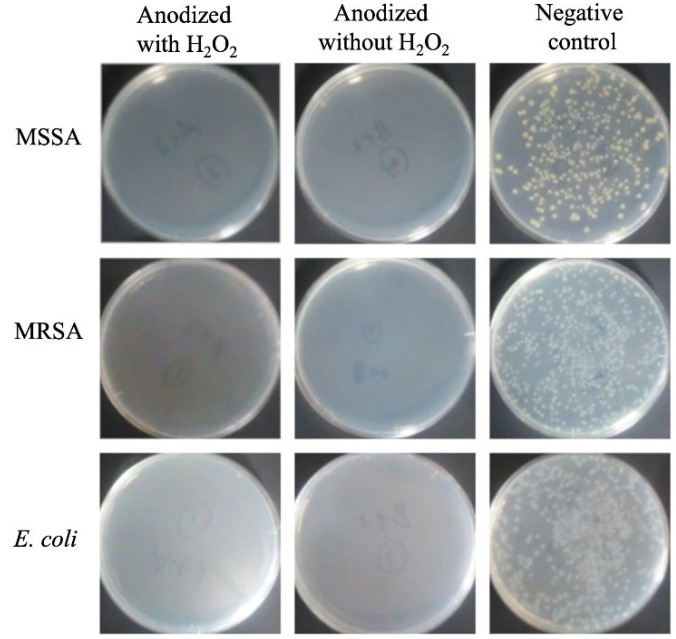
**Bacterial culture tests on TiNbSn alloy anodized with sodium tartrate and glass plate.** The photographs show the results of the culture of MSSA, MRSA, and *E. coli* washout solution after antibacterial tests with anodic oxides with H_2_O_2_ and without H_2_O_2_ and a glass plate under low-intensity ultraviolet light irradiation (0.21 mW/cm^2^) for 8 h. MSSA: Methicillin-sensitive *Staphylococcus aureus*, MRSA: Methicillin-resistant *Staphylococcus aureus*, *E. coli*: *Escherichia coli*.

The photocatalytic and antibacterial performances of anodized TiNbSn alloys prepared in an electrolyte of sodium tartrate acid have been studied [68,69,70,71]. In the development of antibacterial metals, antibacterial metal ions, such as silver and copper, and antibacterial agents, such as iodine and vancomycin, have been used [72,73,74,75]. However, these toxic substances can leak into the bloodstream and cause side effects. TiO_2_ has been reported as a photocatalytic material and has also been used to purify air and water by breaking down redox species using photogenerated charge carriers under illumination corresponding to its band gap energy [76]. In the medical field, TiO_2_ has been reported to have antibacterial properties and anti-tumor effects [77,78,79,80]. When TiO_2_ is irradiated with ultraviolet light, water is decomposed by the photocatalytic effect, and reactive oxygen radicals (ROS) such as hydroxyl radicals (•OH), superoxide anions (O_2_^−^), and hydrogen peroxide (H_2_O_2_) are generated [76]. ROS have been reported to exert antibacterial and antitumor effects by destroying the structure of bacteria and tumor cells [81,82]. In contrast, TiO_2_ is considered a stable and safe substance and is used as an additive in food and pharmaceuticals [83]. TiO_2_-coated biocompatible alloys could be an ideal technology because of their inherent safety and stability, in addition to its antibacterial performance under ultraviolet light irradiation. The photocatalytic activity of the anodized TiNbSn alloy prepared with sodium tartrate with and without H_2_O_2_ was demonstrated. The anode oxides showed a porous microstructure and well-crystallized TiO_2_ with anatase and rutile structures, regardless of the addition of H_2_O_2_ to the electrolyte; the anodic oxide on the TiNbSn alloy prepared with the H_2_O_2_-added electrolyte showed abundant •OH formation and photocatalytic activity under ultraviolet light irradiation, and there was no difference between the two types of anodic oxides (Figure 12). In antibacterial tests, there was no significant difference between the anodic oxides on the TiNbSn alloys with and without H_2_O_2_, and both anodic oxides on the TiNbSn alloys exhibited robust antibacterial activity (Figure 13). This suggests that anodic oxides on TiNbSn alloys are promising biomaterials with low Young’s moduli and antibacterial performances.

## 6. Future Research Development

In this review, the authors describe the effect of TiO_2_, prepared by the anodic oxidation of TiNbSn alloys with acetic acid, sulfuric acid, and sodium tartrate to improve the osseointegration and antibacterial effects due to photocatalytic activity. Efforts to develop high-performance titanium alloys with low Young’s modulus, corrosion resistance, and high biocompatibility that are promising for clinical applications have been reported [84,85,86,87,88,89,90]. Among the new high-performance titanium alloys, TiNbSn alloys, which combine low Young’s modulus with high strength, have already achieved clinical applications. The mid-term results of a clinical trial of a TiNbSn alloy hip prosthesis are reported, with good results in terms of the safety and suppression of stress shielding [17]. In contrast, there are still issues to be resolved to achieve consistent anodizing on large-scale orthopedic prostheses. Compared to anodic oxidation of small TiNbSn alloys, larger electrolytic bath equipment is required for anodic oxidation of large-scale products, and consideration of conditions such as voltage to maintain a constant current is necessary. The stem used in the clinical trial was not anodized, and it is necessary to investigate the possibility of high-quality anodized TiO_2_ in large-scale products such as femoral stem prostheses in the future. Anodization of TiNbSn alloys also imparts a higher exfoliation strength and wear resistance than the Ti6Al4V alloy [70]; therefore, the anodized TiNbSn alloy may also be useful for preventing corrosion in orthopedic implants. Anodized TiNbSn alloys are promising high-performance orthopedic implant materials with high bone affinity and antibacterial properties without compromising their inherently low Young’s modulus.

## 7. Concluding Remarks

The authors presented an in-depth discussion of the properties that make anodized TiNbSn alloys attractive materials for orthopedic surgical applications. The electrochemical properties of anodized TiNbSn alloys were reviewed. We then focused on the surface modification techniques that have been studied to optimize the properties of anodized TiNbSn alloys as orthopedic biomaterials and discussed their osteoconductive performance and potential antibacterial effect due to their photocatalytic performance. Current research advances include the development of equipment and techniques to perform consistent anodizing on large-scale products. As a subject for future research, we propose to adapt the deposition of TiO_2_ by anodic oxidation described in this review to orthopedic devices made of TiNbSn alloys to achieve better long-term results for hip prostheses and other orthopedic implants.

## Data Availability

Data sharing is not applicable to this article.

## Abbreviations

*E. coli*	*Escherichia coli*
EDX	Energy dispersive X-ray spectroscopy
H_2_O_2_	Hydrogen peroxide
MB	Methylene blue
MRSA	Methicillin-resistant *Staphylococcus aureus*
MSSA	Methicillin-sensitive *Staphylococcus aureus*
•OH	Hydroxyl radicals
ROS	Reactive oxygen radicals
SEM	Scanning electron microscopy
TEM	Transmission electron microscope
TiO_2_	Titanium dioxide
XPS	X-ray photoelectron spectroscopy
XRD	X-ray Diffraction
